# Development and validation of a simplified pre-screening model for diabetic foot ulcer identification in diabetic patients

**DOI:** 10.3389/fendo.2026.1847695

**Published:** 2026-05-29

**Authors:** Weidi Wang, Yue Guo, Sining Chen, Wenshi Ou, Qiaoyi Wu

**Affiliations:** 1 Trauma Center and Emergency Surgery Department, The First Affiliated Hospital of Fujian Medical University, Fuzhou, Fujian, China; 2 Hepatopancreatobiliary Surgery Department, National Regional Medical Center, Binhai Campus of the First Affiliated Hospital, Fujian Medical University, Fuzhou, Fujian, China

**Keywords:** albumin, cross-sectional risk identification, diabetic foot ulcer, history of injury, nutritional status

## Abstract

**Background:**

This study aimed to develop a simplified cross-sectional pre-screening tool for diabetic foot ulcer (DFU) using routinely available clinical indicators, with particular focus on the albumin-to-glycated hemoglobin ratio (Alb/HbA1c) as a composite marker of nutritional status and glycemic control.

**Methods:**

We retrospectively analyzed 1,854 hospitalized patients with type 2 diabetes (training set, January 2012–December 2020) and 678 outpatients (validation set, January 2021–December 2025) from a geographically distinct branch of the same hospital system. Demographic characteristics, lifestyle factors, and laboratory parameters were collected. LASSO regression and multivariate logistic regression were used for predictor selection. Model performance was assessed by area under the curve (AUC), calibration curves, Hosmer–Lemeshow test, and Brier score. Risk was visualized using nomograms and heatmaps.

**Results:**

DFU prevalence was 20.9% in the training set and 15.8% in the validation set. Four independent predictors were identified: age (OR = 1.029/year), history of injury (OR = 7.57), alcohol consumption (OR = 0.48, see Discussion for interpretation), and Alb/HbA1c ratio (OR = 0.49). The model showed good discrimination in the training (AUC 0.807, 95% CI 0.790–0.825) and validation sets (AUC 0.817, 95% CI 0.782–0.852), with acceptable calibration (Hosmer-Lemeshow P > 0.05; Brier scores 0.127 and 0.118). Internal validation confirmed stability (optimism-corrected AUC = 0.803). Risk heatmaps revealed synergistic interactions between age and Alb/HbA1c, with injury history amplifying risk across all strata.

**Conclusion:**

This simplified pre-screening model using age, injury history, alcohol consumption, and Alb/HbA1c demonstrates a high negative predictive value, making it suitable for ruling out DFU in resource-limited primary care settings.

## 1 Introduction

Diabetic foot ulcer (DFU) represents one of the most severe chronic complications associated with type 2 diabetes mellitus, exhibiting a global prevalence of approximately 6.3% and a lifetime incidence ranging from 19% to 34% (1).DFU not only leads to a significant decline in patients’ quality of life but is also the primary cause of non-traumatic lower limb amputations, with a 5-year mortality rate exceeding 40% following such procedures (2).Consequently, the early identification of high-risk populations and the implementation of targeted interventions hold significant clinical implications and social value (3).

The development of DFU results from the combined effects of multiple factors, primarily including peripheral neuropathy, peripheral arterial disease, foot deformities, minor trauma, and infection (4).However, existing risk prediction models have largely focused on traditional risk factors, such as glycemic control (HbA1c), vibration perception threshold, and dorsalis pedis pulse (5, 6), while insufficient attention has been paid to nutritional status. Serum albumin serves as a classic indicator of nutritional status and inflammatory burden; hypoalbuminemia has been confirmed to be closely associated with delayed wound healing, increased infection risk, and poor prognosis (7).In recent years, some scholars have proposed that the albumin-to-HbA1c ratio (Alb/HbA1c) may integrate dual information regarding nutritional reserves and long-term glycemic exposure, thereby reflecting a systemic frailty phenotype relevant to DFU (8).

A history of prior foot ulceration is a powerful, yet often underestimated, predictor of DFU recurrence. Epidemiological data indicate that approximately 40% of individuals with DFU experience recurrence within one year of healing, and 60% within three years (1).Repeated tissue trauma can alter the biomechanical properties of the foot, leading to focal pressure abnormalities. Furthermore, scar tissue is often poorly vascularized, resulting in impaired healing capacity following reinjury.

However, the interaction between injury history and nutritional indicators has not been fully explored in prediction models. Additionally, many economically resource-limited settings worldwide, including many mountainous areas in China, still cannot perform complex medical examinations and tests.

To address this gap, we developed a cross-sectional pre-screening model for diabetic foot ulcer using simple, low-cost clinical indicators. This model does not predict future DFU risk; rather, it aims to rule out DFU in patients with a low probability, thereby reducing the burden of medical consultation. For those with a low probability, the model could reduce the burden of medical consultation-such as traveling to advanced centers or undergoing costly foot and vascular exams.

## 2 Methods

### 2.1 Study design and participants

This was a retrospective cross-sectional study using data from the electronic medical record system of inpatients at a general tertiary-care teaching hospital. The study protocol was approved by the hospital’s ethics committee. Given the retrospective design involving de-identified data without additional patient interventions, the hospital’s ethics committee approved the study with a waiver of documented informed consent. The study was conducted in accordance with the principles of the Declaration of Helsinki, ensuring patient privacy protection.

The training cohort enrolled patients with type 2 diabetes mellitus who were hospitalized from January 2012 to December 2020. The validation cohort was obtained from a geographically distinct branch of the same hospital system located in a different city, which predominantly serves a rural population. The training cohort consisted of hospitalized patients to ensure enough outcome events; the validation cohort came from outpatients at a different branch to test generalizability to a less severe population. This validation is therefore described as a temporally and geographically distinct validation within the same health system (quasi-external validation). To guarantee the independence of the datasets, patients with overlapping records in both the main hospital and the branch hospital were excluded.

Inclusion criteria: (1) age ≥18 years; (2) diagnosis of type 2 diabetes mellitus according to the American Diabetes Association criteria (9); and (3) availability of complete demographic data, medical history, and relevant laboratory test results during hospitalization.

Exclusion criteria: (1) foot ulcers of non-diabetic etiology (e.g., venous ulcers, malignancy); (2) history of major lower extremity surgery; (3) presence of diabetic ketoacidosis, hyperosmolar coma, or other acute metabolic complications at admission; (4) active malignancy or ongoing immunosuppressive therapy; (5) severe chronic kidney disease (eGFR < 30 mL/min/1.73 m^2^) or severe liver disease (Child-Pugh class C), severe liver disease or chronic kidney disease were excluded because they independently affect serum albumin levels and may confound the association between Alb/HbA1c and DFU; (6) pregnancy or lactation.

### 2.2 Outcome definition

The primary outcome was a confirmed diagnosis of DFU during hospitalization or an outpatient visit, diagnosed according to the International Working Group on the Diabetic Foot criteria (10). The primary outcome was defined as full-thickness skin loss distal to the ankle in patients with diabetes. To standardize diagnosis across the study period (2012–2025), two specialists (WDW, an endocrinologist; and WSO, a wound care specialist) independently reviewed all cases, with disagreements settled by consensus. We used a structured electronic medical record extraction form. For patients who experienced multiple hospitalizations or outpatient visits, the first occurrence of diabetic foot documented in the medical records was selected for analysis.

### 2.3 Data collection and variable definition

Two trained researchers independently extracted data; a random 5% sample was cross-checked, achieving >98% inter-rater agreement. The following variables were collected:

Demographic characteristics: Age, sex, body mass index (BMI). Overweight was defined as BMI ≥24 kg/m^2^ according to Asian standards (11).

Lifestyle factors: Smoking history (current or former smoker); alcohol consumption history (current or former drinker, ≥6 months of weekly alcohol consumption). History of injury: documented foot trauma within the previous six months, including but not limited to skin ulcers caused by trauma (e.g., cuts, bruises, puncture wounds, or blister formation).

Laboratory parameters included white blood cell count (WBC, ×10^9^/L), hemoglobin A1c (HbA1c, %), total cholesterol (TC, mmol/L), triglycerides (TG, mmol/L), high-density lipoprotein cholesterol (HDL-C, mmol/L), low-density lipoprotein cholesterol (LDL-C, mmol/L), serum albumin (ALB, g/dL), fasting blood glucose (FBG, mmol/L), D-dimer(μg/mL), and serum potassium (K^+^, mmol/L).

Composite indicator: Albumin-to-HbA1c ratio (Alb/HbA1c), calculated as serum albumin (g/dL) divided by HbA1c (%). All laboratory tests were performed on fasting venous blood collected on the morning after admission, using the first available test result.

### 2.4 Statistical analysis

Data analysis was performed using R 4.5.1 and Python 3.11. Two-sided P<0.05 was considered statistically significant.

Descriptive analysis: Continuous variables were first tested for normality (Shapiro-Wilk test and histograms). Normally distributed data were expressed as mean ± standard deviation, with between-group comparisons using independent sample t-tests; non-normally distributed data were expressed as median (interquartile range), with between-group comparisons using Mann-Whitney U tests. Categorical variables were expressed as frequencies (percentages), with between-group comparisons using χ^2^ tests or Fisher’s exact tests.

Nonlinearity testing: Restricted cubic splines (4 knots: 5th, 35th, 65th, and 95th percentiles) were used to test linear relationships between continuous variables and DFU risk (12).If P for nonlinearity <0.05, clinically reasonable cut-points were selected to convert to categorical variables based on spline plots.

Variable screening and model construction: First, LASSO regression (10-fold cross-validation) was applied in the training set for preliminary screening of predictors. The λ value corresponding to the minimum cross-validated error (λ.min) and the largest λ within one standard error of the minimum (λ.1se) were recorded. To balance model parsimony with predictive performance, we selected λ.1se as the optimal penalty parameter. This approach reduces overfitting and facilitates external generalizability (13).Then, variables screened by LASSO were included in a multivariate logistic regression model, and the backward stepwise method was used to determine final predictors, calculating odds ratios (OR) and their 95% confidence intervals (CI). This two-stage approach was adopted to manage multicollinearity initially through penalized regression and subsequently derive a clinically parsimonious set of predictors. However, this hybrid strategy may introduce selection bias and is acknowledged as a potential limitation. Variance inflation factors (VIF) were used to assess multicollinearity, with VIF>5 indicating presence of collinearity.

Model performance evaluation: Evaluated using the area under the receiver operating characteristic curve (AUC) and its 95% CI (2000 bootstrap resamples) (14). Model calibration was evaluated by plotting calibration curves to visualize the agreement between predicted probabilities and observed outcomes, and by calculating the Hosmer–Lemeshow goodness-of-fit statistic (χ^2^ and P value),Overall predictive accuracy was quantified using the Brier score (15). For internal validation, the bootstrap method with 1000 resamples was used to obtain optimism-corrected estimates of the AUC and Brier score. For quasi-external validation, the regression coefficients from the training set were fixed and applied to the validation set to compute predicted probabilities for each patient; discrimination and calibration were then reassessed using the same procedures.

Formal interaction testing: To evaluate whether the visual interaction between age and Alb/HbA1c observed in heatmaps was statistically significant, we added an interaction term (Age × Alb/HbA1c) to the logistic regression model.

Risk visualization: To facilitate clinical interpretation, a nomogram was constructed based on the final logistic regression model. The nomogram converts the regression coefficient of each predictor into a point scale, and the total points correspond to the predicted probability of DFU. In addition, risk heatmaps were generated using LOESS smoothing to visualize the joint effects of age and Alb/HbA1c on DFU risk, stratified by history of injury and alcohol consumption (16).

## 3 Result

### 3.1 Population characteristics

**Figure 1:**
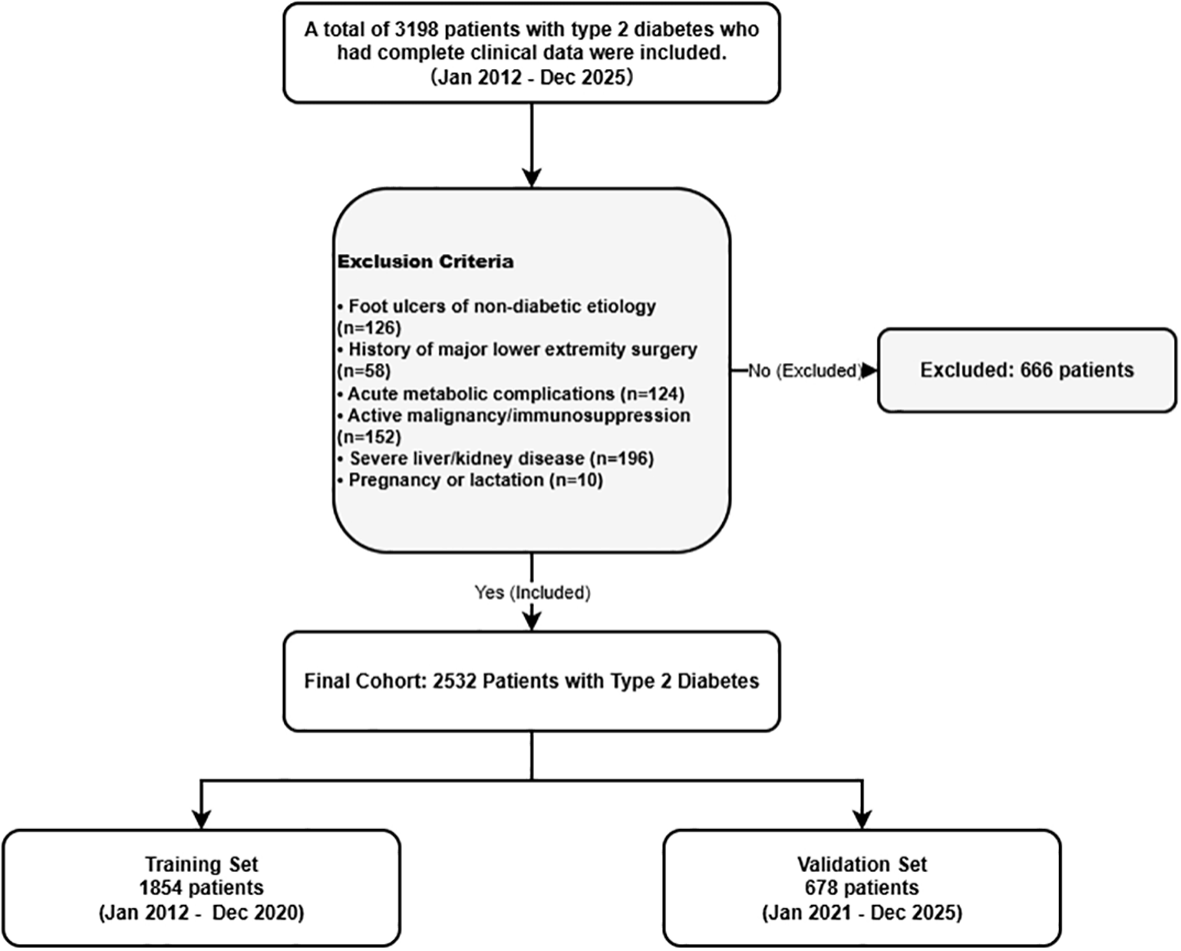
Flow chart of patient selection.

We retrospectively analyzed 1,854 hospitalized patients with type 2 diabetes (training set, January 2012-December 2020) and 678 outpatients from a different city as the validation set (January 2021-December 2025) (**Figure 1**). The prevalence of DFU was 20.9% (388/1854) in the training set and 15.8% (101/678) in the validation set. Baseline characteristics of the two cohorts are summarized in **Table 1**.

**Table 1:** Demographic and clinical baseline data between DFU group and non-DFU group (training set).

Variable	Non-DFU group (n=1466)	DFU group (n=388)	P value
Age (years)	71.00 (62.00, 78.00)	74.00 (67.00, 82.00)	<0.001
Male	1008 (68.8%)	238 (61.3%)	0.0068
Female	458 (31.2)	150 (38.7%)	
BMI (kg/m^2^)	24.4 ± 3.5	24.1 ± 3.2	0.172
Overweight	931 (63.5%)	260 (67.0%)	0.222
alcohol consumption history	530 (36.2%)	76 (19.6%)	<0.001
Smoking history	547 (37.3%)	93 (24.0%)	<0.001
Injury history	64 (4.4%)	104 (26.8%)	<0.001
HbA1c ≥ 6.5 (%)	473 (32.3%)	66 (17.0%)	<0.001
Alb ≥ 35 (g/dL)	867 (59.1%)	247 (63.7%)	0.1192
Alb/HbA1c ratio	5.12 ± 1.43	4.45 ± 1.43	<0.001
WBC (×10^9^/L)	7.00 ± 3.90	9.16 ± 4.88	<0.001
D-dimer (ug/mL)	1.33 ± 1.87	1.45 ± 1.59	0.1649
Potassium ≥ 4 (mmol/L)	764 (52.1%)	253 (65.2%)	<0.0001
Creatinine (mmol/L)	111.08 ± 150.24	111.68 ± 147.06	0.9439
TC (mmol/L)	4.05 ± 1.53	3.99 ± 1.28	0.5108
HDLC>1 (mmol/L)	138 (35.6%)	650 (44.3%)	0.0023
LDLC <2.6 (mmol/L)	213 (54.9%)	862 (58.8%)	0.1845

Data are expressed as number (%), mean ± SD.

BMI, Body Mass Index; Alb, albumin; TC, total cholesterol; HDLC, high density lipoprotein cholesterol; LDLC, low-density lipoprotein cholesterol.

#### 3.1.1 Variable transformation

**Figure 2:**
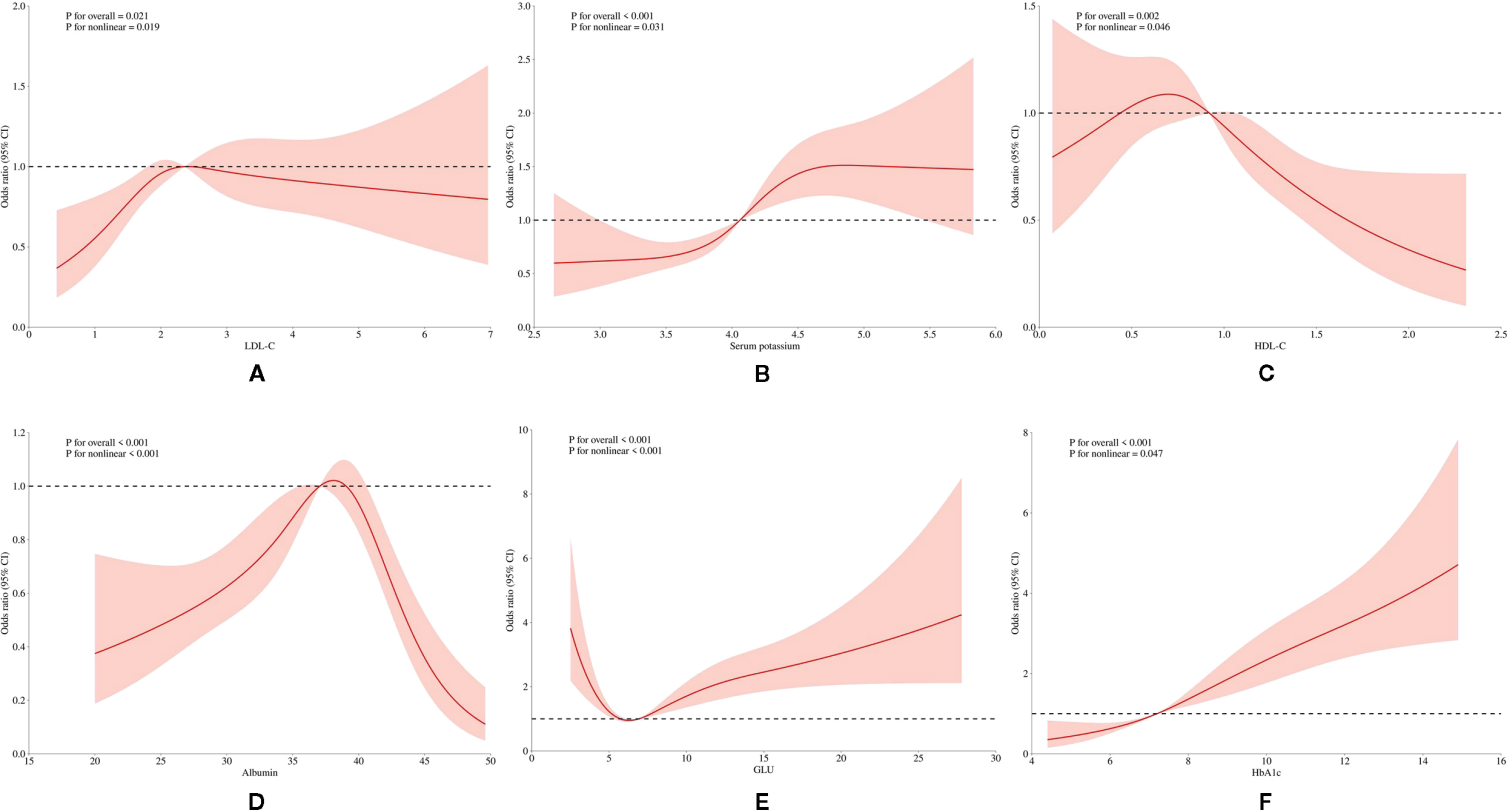
Associations of six laboratory parameters with diabetic foot ulcer risk. Panels **(A)** through **(F)** illustrate the relationships between DFU risk and **(A)** LDL-C, **(B)** serum potassium, **(C)** HDL-C, **(D)** ALB, **(E)** GLU, and **(F)** HbA1c, respectively.

RCS curves were used to test for linear relationships between continuous variables and the outcome variable. Serum potassium, HbA1c, FBG, ALB, HDL-C, and LDL-C exhibited nonlinear associations with the outcome. Accordingly, clinically relevant cut-points were selected to convert these variables into categorical format. HDL-C > 1.0 mmol/L was defined as the optimal level based on the Chinese Guidelines for Lipid Management (2024), with levels below this threshold considered indicative of increased cardiovascular risk (17). LDL-C < 2.6 mmol/L was defined as the ideal level according to the Chinese Guidelines for Lipid Management (2024), representing the target for low-risk populations (18). The RCS plots are presented in **Figure 2**.

Patients with DFU were older than those without DFU (74.1 ± 11.2 vs. 68.9 ± 12.3 years, P < 0.001) and included a slightly higher proportion of females (47.2% vs. 39.7%, P = 0.016). A history of foot injury was markedly more prevalent in the DFU group (32.2% vs. 5.4%, P < 0.001). Compared with the non-DFU group, patients with DFU had significantly lower serum albumin levels (3.89 ± 0.52 vs. 4.12 ± 0.48 g/dL, P < 0.001), higher HbA1c levels (8.62 ± 1.88% vs. 8.28 ± 1.79%, P = 0.002), and consequently a lower albumin-to-HbA1c ratio (4.45 ± 1.43 vs. 5.12 ± 1.43, P < 0.001). In addition, the DFU group exhibited higher WBC counts, lower HDL-C levels, and lower estimated glomerular filtration rates, all of which reached statistical significance (**Table 1**).

### 3.2 Variable screening and multivariate analysis

**Figure 3:**
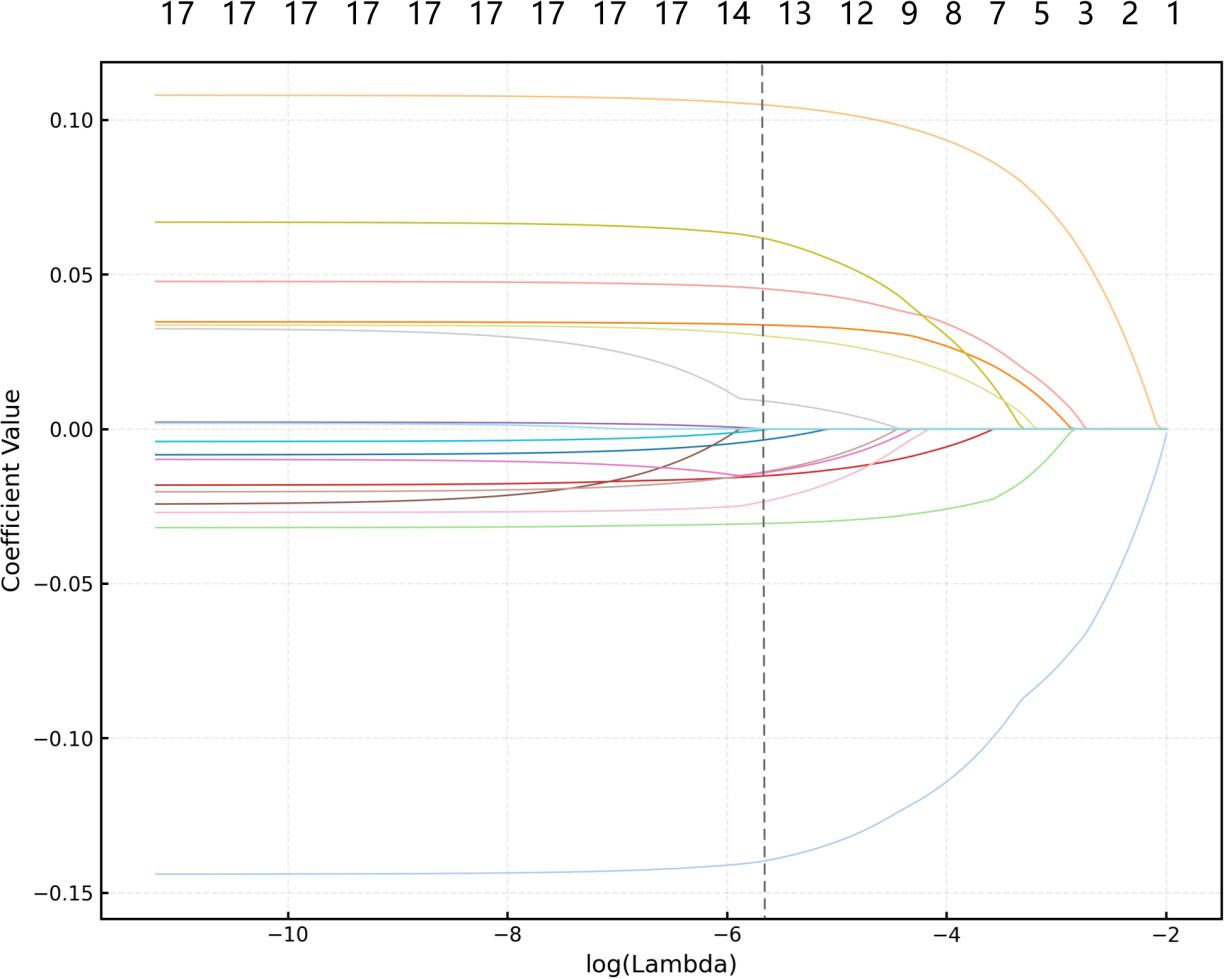
The regression coefficient plot of factors by LASSO. The horizontal axis shows the log of the penalty parameter λ. The vertical axis shows the estimated coefficient values. Each colored line represents one predictor variable, as indicated in the legend. The vertical dashed line marks the optimal λ selected by the one-standard-error rule (lambda.1se).

**Figure 4:**
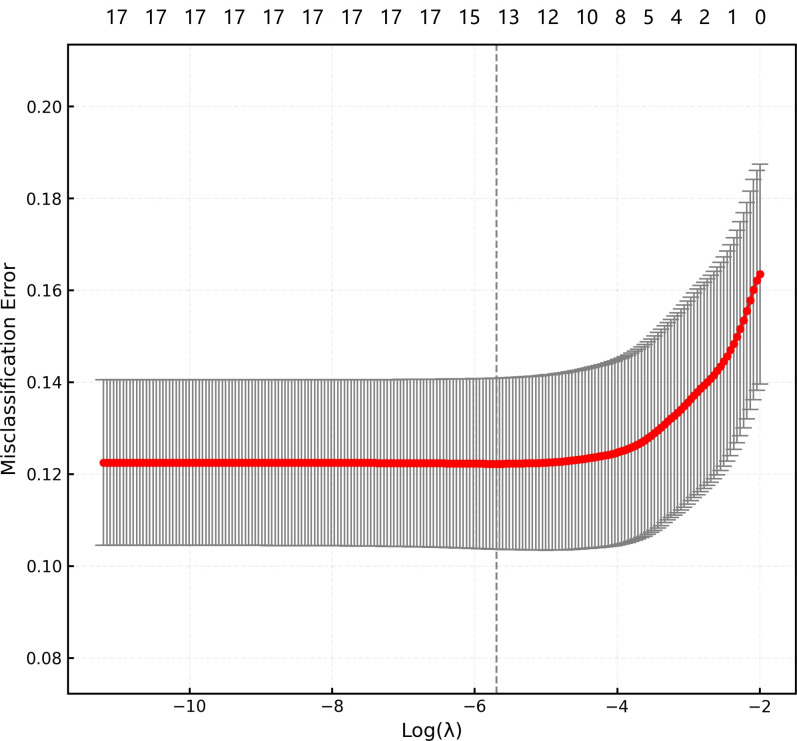
The cross-validation error curve. The x-axis shows log(λ), the penalty parameter; the y-axis shows the binomial deviance (cross-validation error). The red dots represent the average cross-validation error for each λ value, and the error bars represent ±1 standard error. The dotted line indicates the maximum λ value that is one standard error away from the minimum value (lambda.1se). This was selected for model simplicity, resulting in 13 non-zero coefficients.

LASSO regression analysis can effectively handle multicollinearity by reducing the coefficients of relevant predictors and setting some of them to zero. This method generates a more concise and stable model, reduces overfitting, and improves generalization. Therefore, we use LASSO as the initial screening tool to select the most relevant predictors from a large number of related clinical variables. At the selected λ.1se (0.00337, log λ ≈ –5.69), the LASSO regression retained 13 variables with non-zero coefficients (**Figure 3**). For comparison, the λ.min value (0.00129, log λ ≈ –6.65) would have retained 16 variables. The more parsimonious λ.1se model was chosen to enhance generalizability and to avoid overfitting: age, history of injury, alcohol consumption history, Alb/HbA1c, WBC, sex, smoking history, serum potassium, FBG, albumin, HDL-C, TC, and HbA1c (**Figures 3**, **4**). Including the above variables in multivariate logistic regression and using backward stepwise method, four independent predictors were finally determined (all P < 0.05).

The 13 predictors screened by LASSO regression were subsequently entered into multivariate logistic regression analysis (backward stepwise) using R software. The results showed that age, history of injury, alcohol consumption history, and Alb/HbA1c ratio were risk factors for DFU in patients with diabetes (**Table 2**). The final simplified model expression was: Logit(P) = -4.386 + 0.0287×Age - 0.7371×Alcohol consumption + 2.0247×History of injury - 0.7118×Alb/HbA1c.

**Table 2:** Results of multivariate logistic regression analysis.

Variable	β	SE	Wald χ^2^	OR (95% CI)	P value
Age	0.0287	0.006	4.941	1.029 (1.017 ~ 1.041)	<0.001
Injury history	2.0247	0.194	10.434	7.57 (5.32 ~ 10.77)	<0.001
Alcohol consumption history	-0.7371	0.190	-3.873	0.48 (0.33 ~ 0.70)	<0.001
Alb/HbA1c	-0.7118	0.055	-12.874	0.49 (0.44 ~ 0.55)	<0.001

CI, confidence interval. OR, Odds ratio.

A sensitivity analysis excluding history of injury was performed to evaluate the robustness of the remaining predictors. The three-variable model (age, alcohol consumption, Alb/HbA1c) yielded an AUC of 0.745 (95% CI: 0.724–0.776) in the training set, demonstrating that the other variables retained meaningful discriminatory power even without the strongest predictor (**Supplementary Figure 1**).

In this study, variance inflation factors were used to analyze multicollinearity between variables. All variance inflation factors (VIF) were less than 10, indicating no multicollinearity problem among variables (**Table 3**).

**Table 3:** Variance inflation factors for predictor variables.

Characteristic variable	VIF value	Degree of collinearity
Age	4.25	Low
Alb/HbA1c	3.36	Low
Alcohol consumption history	2.39	Low
Injury history	1.13	Very low

VIF, variance inflation factor. All variance inflation factors were below 5, indicating no significant multicollinearity concerns.

### 3.3 Model performance and validation

**Figure 5:**
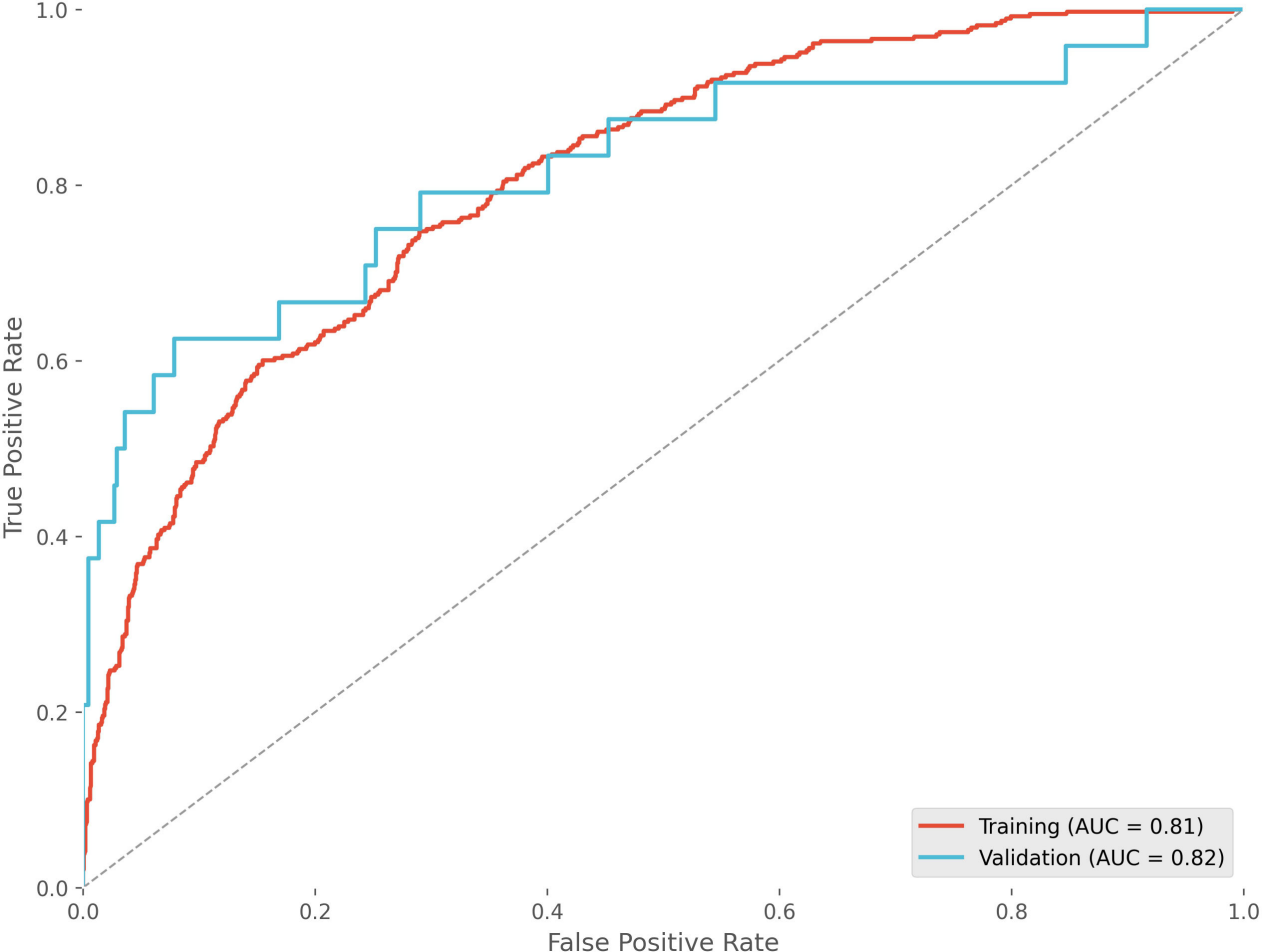
ROC curves of the diabetic foot ulcer risk model. The area under the curve (AUC) was 0.807 [95% confidence interval (CI): 0.790–0.825] for the training set (blue line) and 0.817 (95% CI: 0.782–0.852) for the validation set (red line).

**Figure 6:**
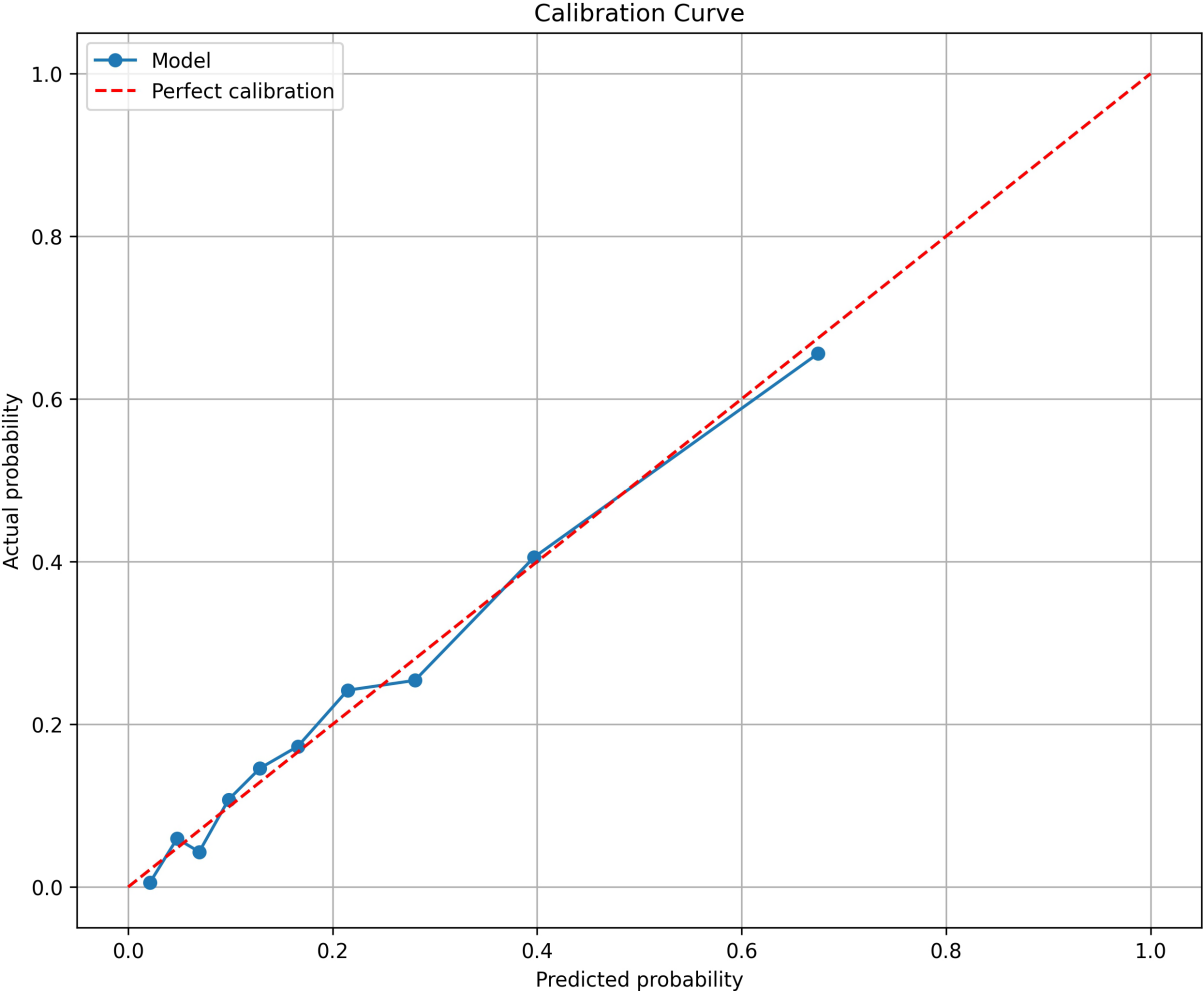
Calibration curves for the diabetic foot ulcer prediction model. The dashed diagonal line represents perfect calibration; the solid line shows the observed performance of the model. The Hosmer-Lemeshow test yielded a P-value of 0.233, indicating no significant deviation from perfect fit. The calibration slope was 0.96 (ideal 1) and the calibration intercept was -0.08 (ideal 0), indicating excellent agreement between predicted and observed risks without systematic over-or under-prediction.

In the training set, the model demonstrated good discrimination with an area under the curve of 0.807 (95% CI: 0.790–0.825) (**Figure 5**). The calibration curve closely approximated the diagonal (**Figure 6**), supported by a non-significant Hosmer–Lemeshow test (χ^2^ = 10.47, P = 0.233), indicating no substantial deviation between predicted probabilities and observed outcomes. The Brier score was 0.127, lower than that of the null model (0.165), consistent with adequate model calibration. After internal validation using 1000 bootstrap resamples, the optimism-corrected AUC was 0.803 and the Brier score was 0.129, confirming model stability.

In the quasi-external validation cohort, the model achieved an AUC of 0.817 (95% CI: 0.782–0.852) (**Figure 5**), with a Hosmer–Lemeshow test P-value of 0.312 and a Brier score of 0.118. Using the optimal cut-off value of 0.192 determined by Youden’s index, the model yielded a sensitivity of 74.7% and a specificity of 70.5% in the training set. In the validation set, sensitivity and specificity were 79.2% and 70.8%, respectively, with a negative predictive value of 94.8% (**Table 4**). For the quasi-external validation cohort, the calibration slope was 1.02 and the calibration intercept was -0.15, further confirming good calibration with a slight tendency toward modest risk underestimation (**Supplementary Figure 2**).

**Table 4:** Performance metrics of the model in training and validation sets.

Metric	Training set	Validation set
AUC (95% CI)	0.807 (0.790–0.825)	0.817 (0.782–0.852)
Hosmer-Lemeshow χ^2^ (P)	10.47 (0.233)	9.84 (0.312)
Brier score	0.127	0.118
Optimal cut-off value	0.192	0.192
Sensitivity (%)	74.7	79.2
Specificity (%)	70.5	70.8
Positive predictive value (%)	39.8*	33.1*
Negative predictive value (%)	91.4*	94.8*

AUC, area under the receiver operating characteristic curve; The optimal cut-off value was determined by Youden’s index.

*All P-values for the Hosmer–Lemeshow test were >0.05, indicating good calibration.

### 3.4 Risk visualization

**Figure 7:**
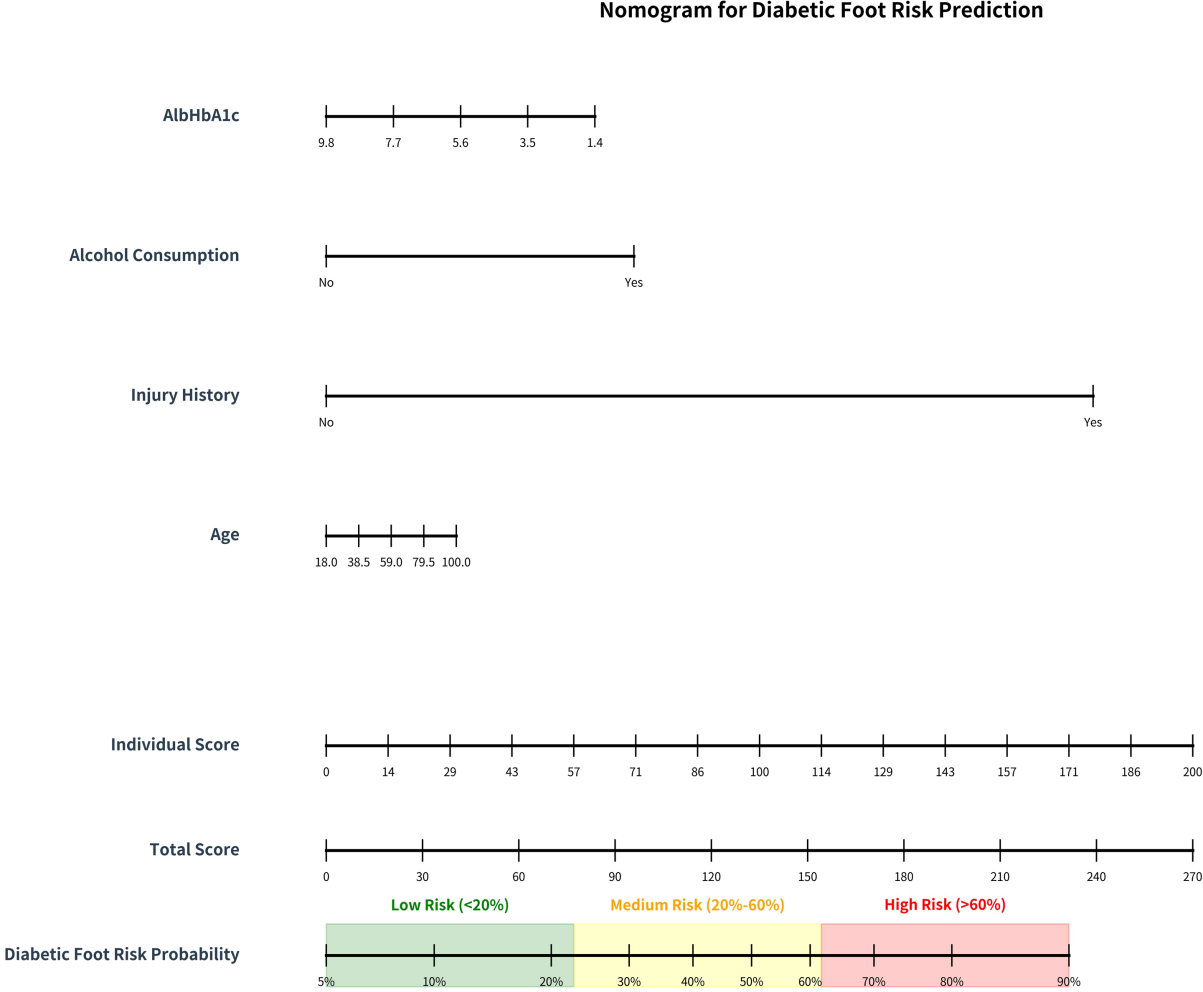
Nomogram for DFU simplified pre-screening model. To use the nomogram, draw a vertical line upward from each predictor variable to the “Points” scale to assign a score. Sum the scores for all variables, locate the total on the “Total Points” axis, and then draw a vertical line downward to obtain the corresponding predicted probability of DFU.

The nomogram (**Figure 7**) translates the regression coefficient of each predictor into a point-based scale, enabling rapid estimation of individualized DFU risk. To facilitate rapid bedside risk estimation, we converted the logistic regression coefficients into an integer point-score system. The conversion was performed by dividing each coefficient by the smallest absolute coefficient (Alb/HbA1c, 0.7118) and multiplying by 10, then rounding to the nearest integer. The total score ranges from 0 to 118. The corresponding DFU probability is approximately: Total score ≤ 30: risk <5%; Total score 31–60: risk 5%–15%; Total score 61–90: risk 15%–30%; Total score ≥ 91: risk >30% (**Supplementary Table 1**).

**Figure 8:**
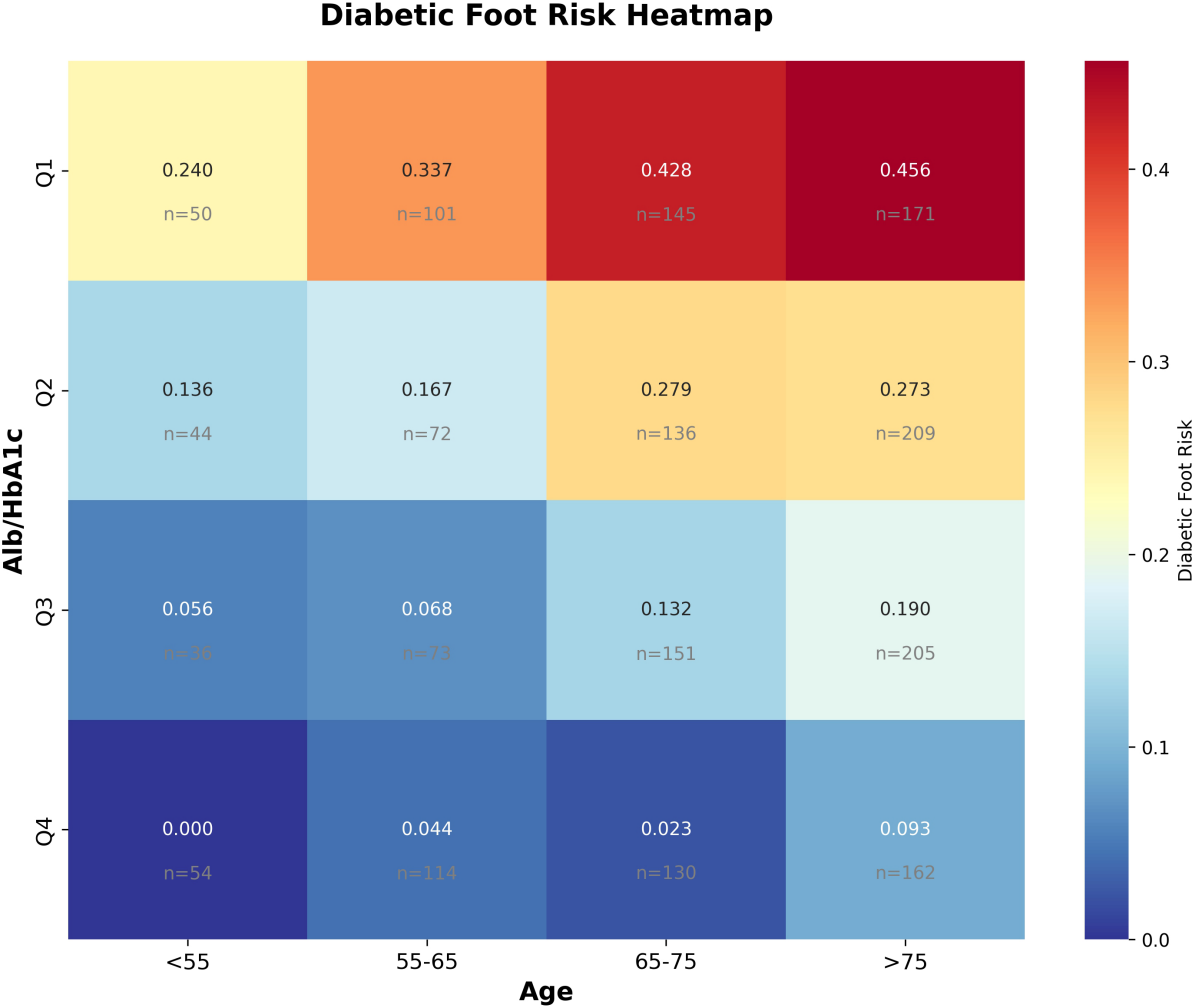
Heatmap visualization of the interaction between age and Alb/HbA1c on DFU risk. The risk gradient becomes steeper at lower Alb/HbA1c levels, indicating that age amplifies risk predominantly under poor nutritional-glycemic control (Predicted outcomes are shown).

Risk heatmaps revealed interactive effects between predictors. A significant interaction was observed between Alb/HbA1c and age: at low Alb/HbA1c levels, the risk-enhancing effect of age was more pronounced (**Figure 8**), whereas at high Alb/HbA1c levels, the influence of age was attenuated. Added interaction term (Age × Alb/HbA1c) to logistic regression β = 0.009, OR = 1.009 (95% CI: 1.000–1.018). The interaction term was statistically significant (P < 0.001) (see **Supplementary Table 2**). Both stratified analysis and heatmap data confirm that Alb/HbA1c has a negative association against diabetic foot complications, but this negative association is attenuated in older patients. To validate the interaction effect, stratified logistic regression was performed for each age group: the stratified analysis confirms the interaction effect: the Alb/HbA1c coefficient increases from -0.946 (OR = 0.388) in patients <55 years to -0.635 (OR = 0.530) in patients >75 years, indicating that the negative association of Alb/HbA1c weakens with increasing age (**Supplementary Table 3**).

**Figure 9:**
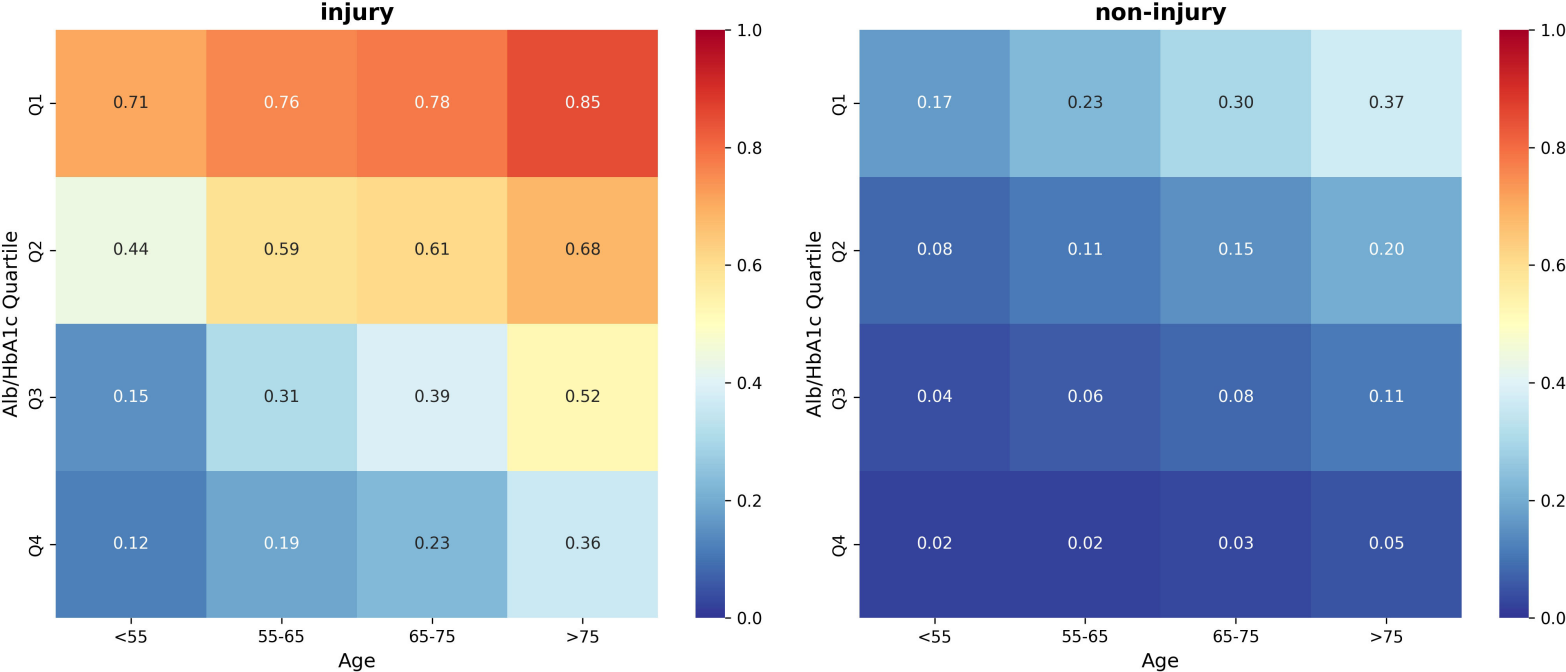
Risk heatmaps for DFU prediction stratified by history of injury. The color gradient represents the predicted probability of DFU based on age (x-axis) and Alb/HbA1c ratio (y-axis), generated using LOESS smoothing. Darker colors indicate higher risk.

**Figure 10:**
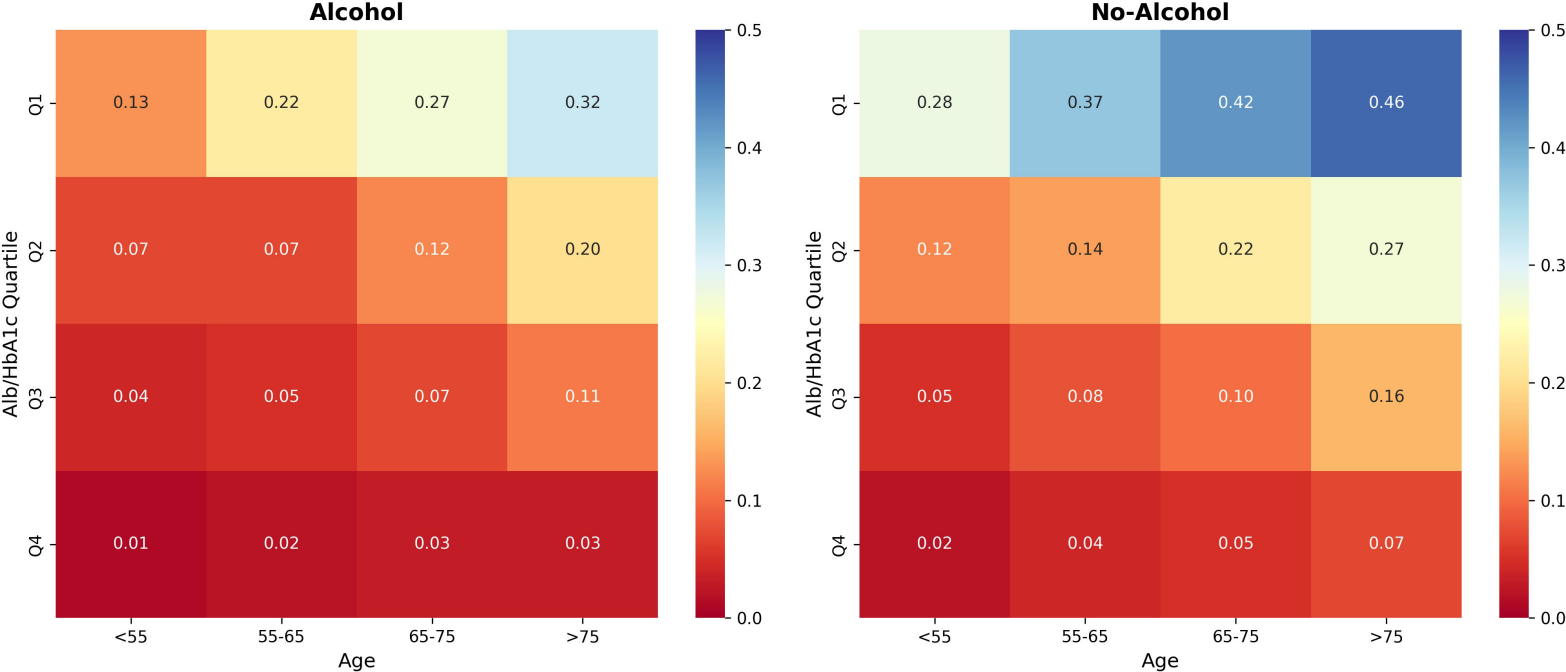
Risk heatmaps for DFU prediction stratified by alcohol consumption history. The color gradient represents the predicted probability of DFU based on age (x-axis) and Alb/HbA1c ratio (y-axis), generated using LOESS smoothing. Darker colors indicate higher risk.

After stratification by injury history, patients with prior injury exhibited a predicted DFU risk exceeding 30% even at relatively high Alb/HbA1c levels. In contrast, among patients without injury history and with an Alb/HbA1c ratio >5.0, the predicted risk was below 10% (**Figure 9**). Alcohol consumption history showed similar patterns across all strata (**Figure 10**).

**Figure 11:**
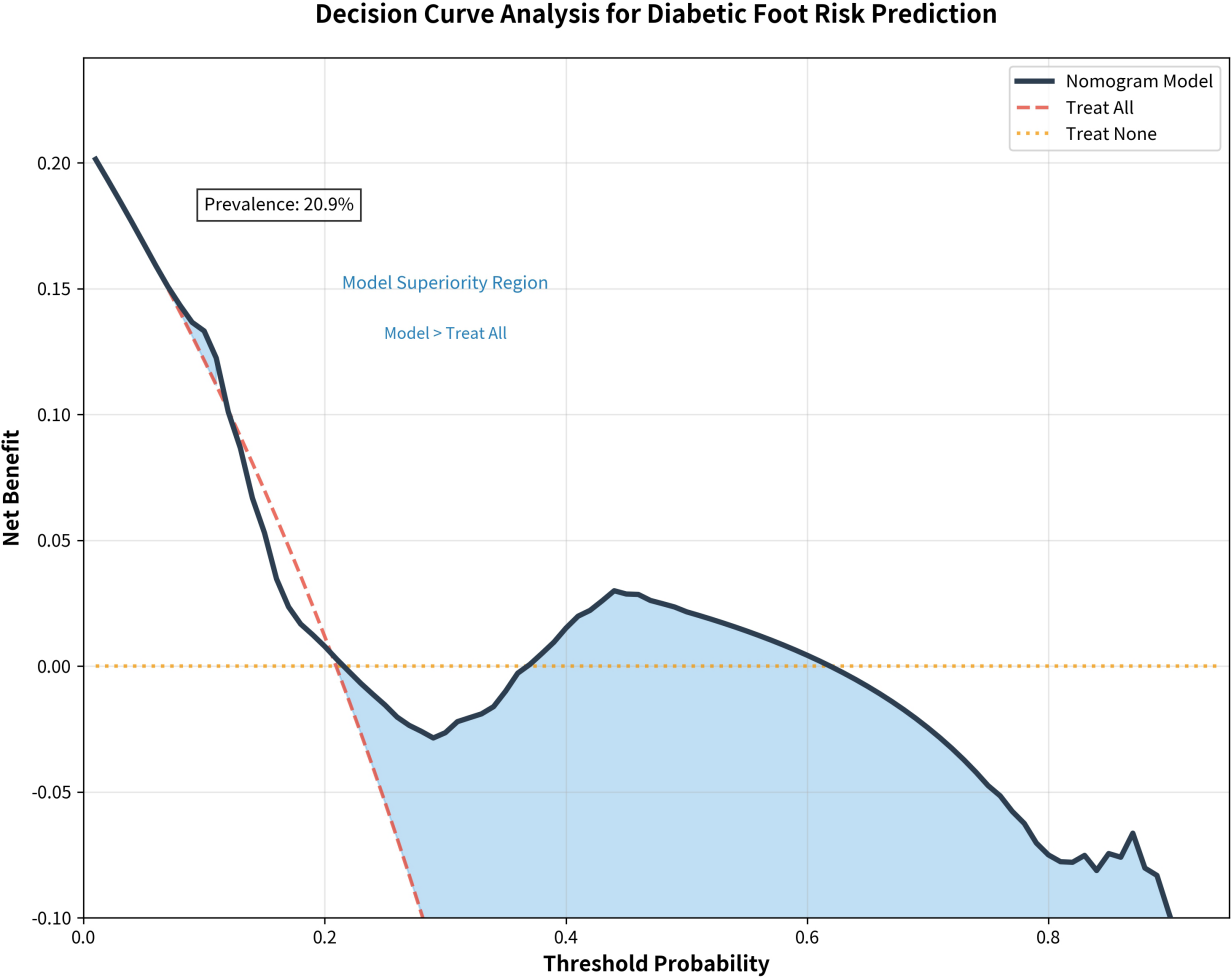
Decision curve analysis for the DFU risk model. The y-axis represents net benefit, and the x-axis represents threshold probability. The model (red line) provides superior net benefit compared with both the “treat-all” and “treat-none” strategies across threshold probabilities ranging from 10% to 40%.

Decision curve analysis showed that the model had a positive net benefit compared with the “treat-all” and “treat-none” strategies within a threshold probability range of roughly 10–40% (**Figure 11**). Outside this range, the net benefit was similar to or lower than the default strategies. Thus, the model is most helpful when clinicians consider an intervention-such as enhanced foot examination or education-for patients whose estimated DFU risk falls between 10% and 40%.

## 4 Discussion

### 4.1 Main findings

This study developed and validated a simplified cross-sectional pre-screening model for DFU using age, injury history, alcohol consumption, and the Alb/HbA1c ratio. We emphasize the following critical clarifications:

1. This is NOT a prospective risk prediction model. Therefore, reverse causality cannot be excluded. Lower albumin, higher WBC, and even injury history may be consequences rather than causes of DFU.
2. The model is NOT a substitute for comprehensive foot examination. It does not include neuropathy, biomechanical deformities, peripheral arterial disease, or plantar pressure assessment - all established DFU determinants.
3. The model is best used to “rule out” DFU in low-risk patients (high negative predictive value) in resource-limited primary care settings where advanced foot exams are not available. Its modest positive predictive value means that positive screens require confirmatory clinical evaluation.

### 4.2 Alb/HbA1c: a composite surrogate marker

The Alb/HbA1c ratio represents a core component of this study. It is viewed as a composite surrogate marker reflecting systemic nutritional-inflammatory reserve and glycemic burden. Low albumin may reflect inadequate nutritional reserves and/or chronic inflammatory consumption; elevated HbA1c indicates prolonged hyperglycemic exposure. A diminished ratio may identify a frailty phenotype where the organism sustains glucotoxicity under nutritional depletion. This interpretation is hypothesis-generating, because albumin and HbA1c can be influenced by factors unrelated to DFU, such as hydration status, anemia, liver disease, and inflammation.

From a pathophysiological perspective, albumin not only maintains plasma colloid osmotic pressure but also facilitates the transport of fatty acids, hormones, and trace elements (e.g., zinc, copper)—all essential for collagen synthesis and epithelial regeneration (19). Hypoalbuminemia inhibits fibroblast proliferation, reduces angiogenesis, and delays wound healing (20). Concurrently, sustained hyperglycemia compromises local circulation and neural function through accumulation of advanced glycation end-products, oxidative stress, and microangiopathy (21). However, these mechanisms are speculative; our data do not directly test causal pathways. Thus, a low Alb/HbA1c ratio may identify a “vulnerable phenotype” wherein foot tissue exists in a subclinically compromised state even in the absence of overt complications, rendering it susceptible to ulceration following minor trauma.

Previous investigations have predominantly focused on single indicators. Shi et al. identified serum albumin as an important risk factor for DFU occurrence using random forest modeling but did not examine its interaction with glycemic control (22). Peng et al. demonstrated that both hypoalbuminemia and elevated HbA1c independently predicted amputation risk in DFU patients, supporting the biological plausibility of their combined assessment (23). The present study extends this concept by formally integrating these parameters into a composite metric specifically for DFU prediction and validating its robustness across temporally independent cohorts.

The Alb/HbA1c ratio showed an independent association with DFU in our multivariate model. We note that in univariate analysis, serum albumin alone was not significantly associated with DFU, and in the multivariate model, HbA1c alone was not an independent risk factor. However, the composite ratio Alb/HbA1c was statistically significant. This finding suggests that the ratio may capture combined information beyond that provided by either component individually. Nevertheless, we did not perform formal model comparisons to demonstrate superiority over albumin or HbA1c alone. Therefore, our results should be interpreted as hypothesis-generating, and future studies are needed to directly compare these models.

### 4.3 History of injury: a strong but proximal indicator

History of injury emerged as the strongest independent predictor of DFU (OR = 7.57), consistent with reported recurrence risks in the literature (24, 25). The risk heatmaps generated in this study further elucidate that injury history substantially amplifies the probability of disease when combined with age and Alb/HbA1c: among individuals with prior injury, DFU risk remained elevated even when metabolic and nutritional parameters were relatively favorable (26). This finding underscores that all patients with a history of foot injury should be automatically classified as extremely high-risk, irrespective of laboratory results, warranting immediate implementation of intensive preventive measures—such as custom orthopedic footwear, regular professional foot examinations, and prompt treatment of any foot lesions (27). However, in this study, “history of injury” was defined as documented foot trauma within the previous six months. This variable therefore represents a recent triggering event rather than a remote history of prior ulcer or amputation. The strong association is clinically plausible, as recent foot trauma can directly lead to soft tissue breakdown, introduce infection, or exacerbate existing microvascular compromise in diabetic patients. Importantly, it can serve as a short-term indicator for identifying the occurrence of DFU, rather than a long-term predictor. A sensitivity analysis demonstrated that after removing injury history, the model still showed moderate discriminative ability (AUC 0.745), confirming the independent value of the other three variables.

### 4.4 Age

Each one-year increase in age was associated with a 2.9% elevated DFU risk, an effect independent of diabetes duration and complications. This likely reflects age-related reductions in skin thickness, microcirculatory function, immune senescence, and heightened vulnerability due to polypharmacy (e.g., anticoagulants) (28).The observed interaction between age and Alb/HbA1c suggests that elderly patients warrant particular attention to the equilibrium between nutritional status and glycemic control.

### 4.5 Alcohol consumption history: associations requiring cautious interpretation

The negative coefficient for alcohol consumption history (OR = 0.48) should not be interpreted as a causal protective effect. Residual confounding (e.g., healthier lifestyle among moderate drinkers, higher socioeconomic status, or reverse causality) or reporting bias likely explains this association. No clinical recommendation regarding alcohol use can be derived from this study.

### 4.6 Clinical utility

Compared with existing DFU prediction models, our approach offers several advantages: 1.Simplicity: using only four routinely available variables, it achieves predictive performance comparable to more complex models. The Seattle model developed by Boyko et al. incorporates monofilament insensitivity, prior amputation, and transcutaneous oxygen pressure; although yielding similar discrimination, certain assessments remain impractical in resource-limited settings (6), 2. Novel composite indicator: first integration of nutrition-glycemic balance (Alb/HbA1c) expands the risk assessment dimension beyond traditional parameters. Our approach offers simplicity (four variables) but sacrifices completeness (no neuropathy, PAD, or biomechanical assessment). The model achieves a high negative predictive value (NPV) of 91.4% in the training set and 94.8% in the validation set, indicating that patients with a low risk score are very unlikely to have DFU. In resource-limited primary care settings where comprehensive foot examinations (neuropathy, biomechanical, or vascular assessments) are unavailable, this high NPV can help avoid unnecessary referrals, reduce healthcare costs, and focus scarce resources on patients with moderate-to-high risk scores. Decision curve analysis showed net benefit only within a threshold probability range of 10–40%, suggesting the model’s clinical usefulness is context-dependent.

### 4.7 Limitations

This study has several important limitations, many of which are inherent to its retrospective cross-sectional design:

1. Reverse causality. Because predictors and outcome were measured within the same hospitalization, we cannot determine whether low albumin, elevated WBC, or even injury history preceded DFU or resulted from it. DFU-related inflammation, infection, and malnutrition could lower albumin and raise HbA1c. Thus, our model identifies associations, not causal risk factors, and should not be interpreted as a prospective prediction tool.
2. Absence of key foot-specific clinical parameters. The model does not include peripheral neuropathy (monofilament, vibration perception), foot deformities (Charcot foot, claw toes), biomechanical measures (plantar pressure), or peripheral arterial disease (ankle-brachial index). Consequently, the model cannot identify patients whose DFU risk is driven primarily by mechanical or vascular factors in the absence of metabolic/nutritional derangements. A low model score should not deter a basic foot inspection.
3. Selection bias. Excluding severe CKD and advanced liver disease may have underestimated the true predictive value of Alb/HbA1c in real-world populations where such comorbidities are common. Additionally, the training cohort comprised only hospitalized patients, who generally have more severe illness than outpatients; this may limit generalizability to primary care settings. The two-stage variable selection process (LASSO followed by stepwise regression) was adopted to balance stability with model parsimony. However, this hybrid approach may introduce model-selection bias and may not be perfectly reproducible in an external dataset. A single, consistent penalized regression framework may be preferable in future work to confirm variable selection.
4. Validation limitations. The validation cohort, although temporally and geographically distinct, originated from the same hospital system. True external validation in an independent multicenter cohort is needed. Meanwhile, the inpatient derivation cohort may overrepresent more severe metabolic derangements, potentially inflating the apparent importance of Alb/HbA1c compared with a purely community-based sample.
5. As noted, the negative association between DFU and alcohol consumption history is almost certainly due to residual confounding or bias.
6. The model’s modest positive predictive value (approximately 33–40%) limits its utility as a rule-in tool. Therefore, we recommend its use only as a pre-screening (rule-out) instrument in resource-limited settings, with confirmatory evaluation for screen-positive cases. In addition, the integer point-score system was not independently validated and may introduce minor imprecision due to rounding; it should be used as a rapid estimation tool rather than a precision calculation.

## 5 Conclusion

This simplified cross-sectional pre-screening model using age, injury history, alcohol consumption, and Alb/HbA1c demonstrates a high negative predictive value, making it suitable for ruling out DFU in resource-limited primary care settings. It may help reduce the burden of unnecessary referrals for patients with a low probability of having DFU. Positive screens require confirmatory clinical foot examination.

## Data Availability

The raw data supporting the conclusions of this article will be made available by the authors, without undue reservation.

## Supplementary material

Supplementary Figure 1 ROC curve for the sensitivity analysis model excluding history of injury. The three-variable model (age, alcohol consumption, and Alb/HbA1c ratio) demonstrated moderate discriminative ability for diabetic foot ulcer in the training set, with an area under the curve of 0.745 (95% CI: 0.724–0.776).

Supplementary Figure 2 Calibration curve for the simplified pre-screening model in the quasi-external validation cohort. Calibration plot assessing the agreement between predicted probabilities and observed outcomes in the quasi-external validation cohort (n=678). The solid line indicates the model’s performance, with a calibration slope of 1.02 and a calibration intercept of -0.15.
