# Supplementary figures and images for: Development and validation of a simplified pre-screening model for diabetic foot ulcer identification in diabetic patients
Source: Front Endocrinol (Lausanne). 2026 May 29;17:1847695. doi: 10.3389/fendo.2026.1847695 (PMC13259758; doi:10.3389/fendo.2026.1847695)

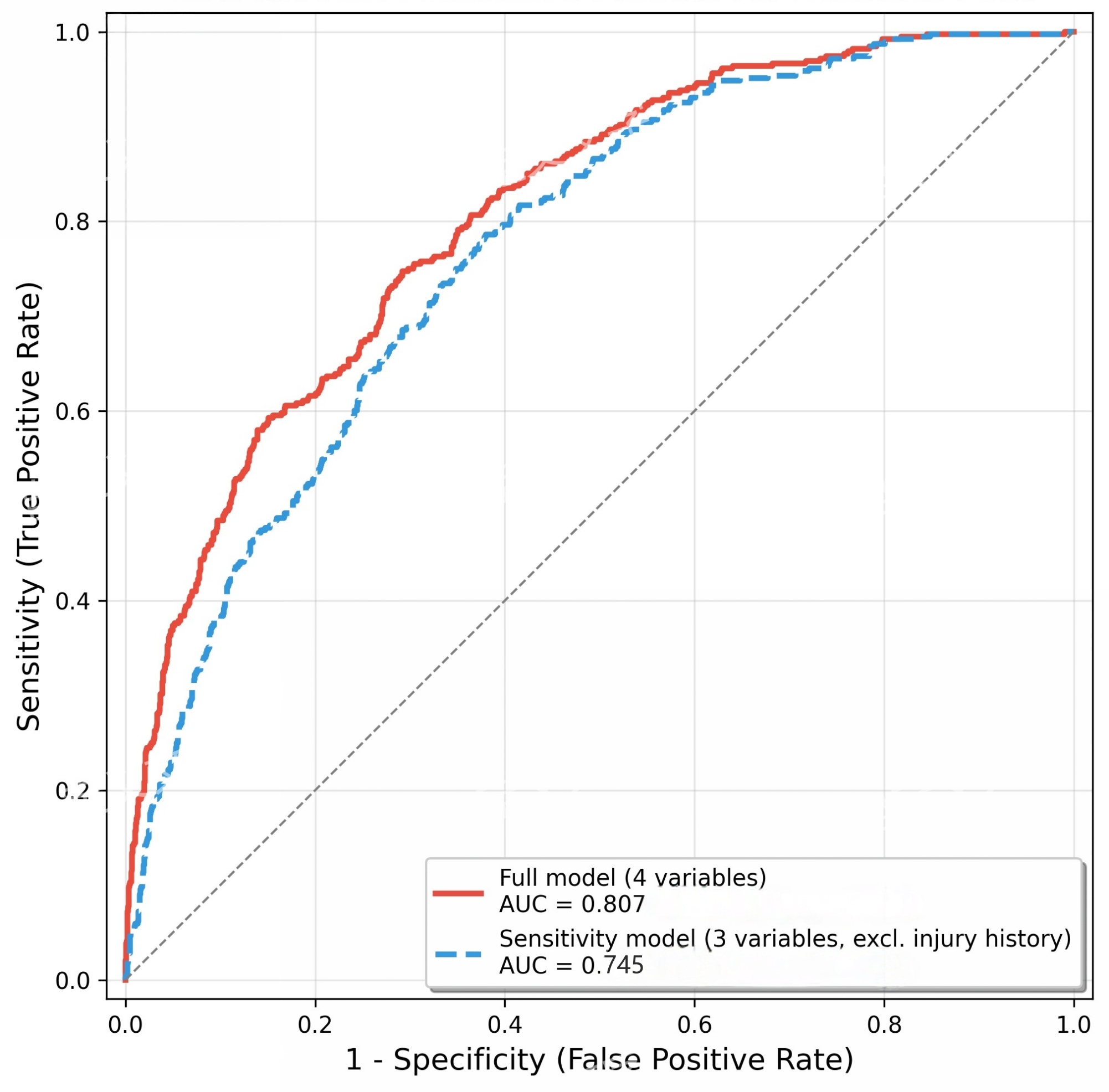

Supplement: Supplementary Figure 1 — ROC curve for the sensitivity analysis model excluding history of injury. The three-variable model (age, alcohol consumption, and Alb/HbA1c ratio) demonstrated moderate discriminative ability for diabetic foot ulcer in the training set, with an area under the curve of 0.745 (95% CI: 0.724–0.776). [file Image1.jpeg]

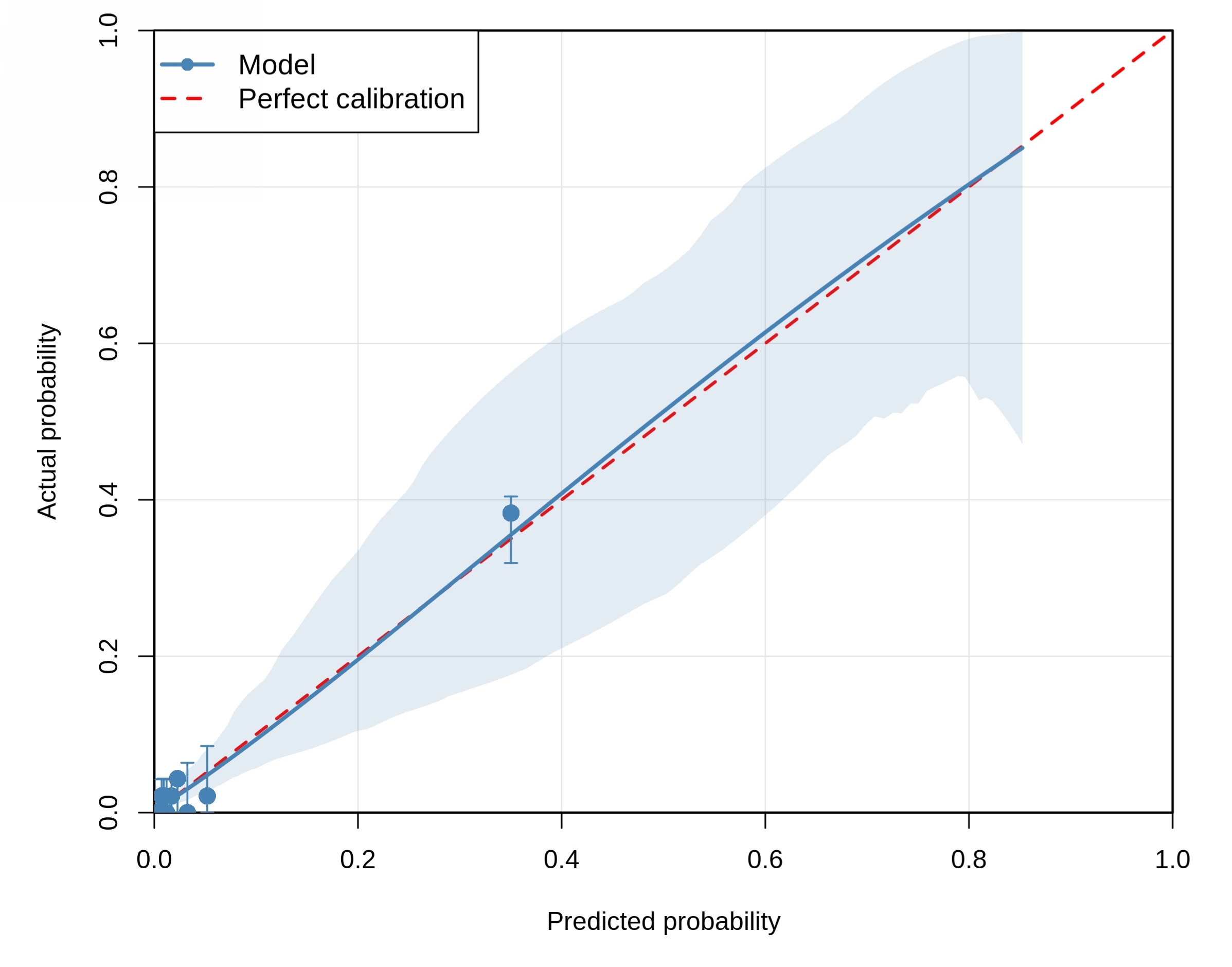

Supplement: Supplementary Figure 2 — Calibration curve for the simplified pre-screening model in the quasi-external validation cohort. Calibration plot assessing the agreement between predicted probabilities and observed outcomes in the quasi-external validation cohort (n=678). The solid line indicates the model’s performance, with a calibration slope of 1.02 and a calibration intercept of -0.15. [file Image2.jpeg]
