# Supplementary material for: Development and validation of a simplified pre-screening model for diabetic foot ulcer identification in diabetic patients
Source: Front Endocrinol (Lausanne). 2026 May 29;17:1847695. doi: 10.3389/fendo.2026.1847695 (PMC13259758; doi:10.3389/fendo.2026.1847695)
Supplement: Supplementary file 3 [file Table1.docx]

| Variable | Category | Points |
| --- | --- | --- |
| **Age** | <60 years | 0 |
|  | 60–69 years | 4 |
|  | 70–79 years | 8 |
|  | ≥80 years | 12 |
| **History of injury (within 6 months)** | No | 0 |
|  | Yes | 28 |
| **Alcohol consumption** | Yes | 0 |
|  | No | 10 |
| **Alb/HbA1c ratio** | >5.0 | 0 |
|  | 4.0–5.0 | 10 |
|  | 3.0–3.9 | 20 |
|  | <3.0 | 30 |
| ****Total points**** |  |  |

Supplementary Table 1. Simplified Point-Score System for Rapid Estimation of DFU Probability.

The total score ranges from 0 to 118. The corresponding DFU probability is approximately:

Total score≤30: risk <5%; Total score 31–60: risk 5%–15%; Total score 61–90: risk 15%–30%; Total score ≥ 91: risk >30%.
