# Supplementary material for: Development and validation of a simplified pre-screening model for diabetic foot ulcer identification in diabetic patients
Source: Front Endocrinol (Lausanne). 2026 May 29;17:1847695. doi: 10.3389/fendo.2026.1847695 (PMC13259758; doi:10.3389/fendo.2026.1847695)
Supplement: Supplementary file 4 [file Table2.docx]

| **Variable** | **Coefficient (β)** | **SE** | **OR (95% CI)** | **z-value** | **P-value** |
| --- | --- | --- | --- | --- | --- |
| Intercept | -1.666477 | 0.076165 | 0.188911 (0.162715, 0.219326) | -21.8800 | <0.001 |
| Age (years) | 0.041835 | 0.006538 | 1.042722 (1.029446, 1.056169) | 6.3989 | <0.001 |
| Alb/HbA1c | -0.730134 | 0.053491 | 0.481844 (0.433886, 0.535104) | -13.6497 | <0.001 |
| Age × Alb/HbA1c | 0.008596 | 0.004531 | 1.008633 (0.999715, 1.017630) | 1.8972 | <0.001 |

Supplementary Table 2. Interaction Analysis: Age × Alb/HbA1c in DFU Prediction.

Age: = 0.0418 (p<0.001). The positive coefficient indicates that age is a risk factor for β diabetic foot complications – older patients have higher risk. Alb/HbA1c: = -0.7301 (p<0.001). The negative coefficient indicates a significant protective β effect of higher Alb/HbA1c ratio against diabetic foot complications.
