# Supplementary material for: Development and validation of a simplified pre-screening model for diabetic foot ulcer identification in diabetic patients
Source: Front Endocrinol (Lausanne). 2026 May 29;17:1847695. doi: 10.3389/fendo.2026.1847695 (PMC13259758; doi:10.3389/fendo.2026.1847695)
Supplement: Supplementary file 5 [file Table3.docx]

| Age Group | Alb/HbA1c β | Alb/HbA1c OR | P-value |
| --- | --- | --- | --- |
| <55 | -0.9457 | 0.3884 | <0.001 |
| 55-65 | -0.8223 | 0.4394 | <0.001 |
| 65-75 | -0.7099 | 0.4917 | <0.001 |
| >75 | -0.6353 | 0.5298 | <0.001 |

Supplementary Table 3. Analysis and validation based on age stratification.

The stratified analysis confirms the interaction effect: the Alb/HbA1c coefficient increases from -0.946 (OR=0.388) in patients <55 years to -0.635 (OR=0.530) in patients >75 years, indicating that the protective effect of Alb/HbA1c weakens with increasing age.
